# Genotype Distribution and Antibiotic Susceptibility Pattern of Clinical Isolates of Group B *Streptococcus* in a Tertiary Care Hospital in Puducherry, South India

**DOI:** 10.1155/2023/9910380

**Published:** 2023-03-08

**Authors:** A. V. Sangeetha, Sheela devi, Anandhalakshmi Subramanian, Mary Daniel, Perumal Anandh

**Affiliations:** ^1^Laboratory Division, Central Leprosy Teaching and Research Institute, Chengalpattu 604001, Tamil Nadu, India; ^2^Department of Microbiology, Pondicherry Institute of Medical Sciences, Puducherry 605014, India; ^3^Department of Microbiology and Parasitology, College of Medicine, King Khalid University, Abha 62521, Saudi Arabia; ^4^Department of Obstetrics and Gynecology, Pondicherry Institute of Medical Sciences, Puducherry 605014, India; ^5^Department of Microbiology, Manakula Vinayagar Medical College, Kalitheerthalkuppam, Puducherry, India

## Abstract

**Background:**

*Streptococcus agalactiae* apart from being a colonizer in the genital region is also associated with several other invasive infections in all age groups. With the varied distribution of serotypes across different regions of the world, universal vaccination is also unattainable. However, in India, the knowledge of group B *Streptococcus* (GBS) genotype distribution is deficient. Thus, this study was initiated to add data on this aspect. *Methodology*. A cross-sectional study was conducted using isolates of group B *Streptococcus* from all clinical specimens. Along with that, the clinical specimen type and the antibiotic resistance profile of the isolates were correlated with the genotypes recognized through a multiplex PCR assay.

**Results:**

Among the 86 isolates subjected to multiplex PCR for genotype identification, five genotypes were identified with genotype Ib as the predominant one (34.9%), followed by III (20.9%), II (16.3%), Ia (12.7%), and V (11.6%).

**Conclusion:**

The results demonstrated a correlation of types Ib and III with vaginal colonization and type II with urine specimens in the current study. This preliminary study exhibited the distribution of common genotypes and their antibiotic resistance profiles in various GBS isolates. However, multiple studies across the country with larger sample sizes are needed to validate these findings.

## 1. Introduction

*Streptococcus agalactiae* is associated with a wide variety of clinical features like urinary tract infection, wound infection, and vaginal colonization, leading to premature rupture of membranes and preterm labor, neonatal sepsis, arthritis, osteomyelitis, and septicemia in elderly individuals with other medical comorbid conditions [1]. Group B *Streptococcus* has nine serotypes based on capsular polysaccharides. The distribution of these serotypes among various clinical infections and geographical locations is different, necessitating the need for typing methods [2–4].

The routine serotyping methods available are coagglutination, latex agglutination, counter-current immunoelectrophoresis, capillary precipitation, immunoprecipitation, enzyme immunoassay, and fluorescence microscopy [1, 5, 6]. However, they are labor-intensive and require high titer antisera which is not cost effective. Moreover, there are certain strains that are nontypeable by these conventional methods [1]. Thereby, molecular methods to genotype this organism acts as a feasible approach since the sequences of gene clusters in all nine serotypes have been published [7].

Several such studies are carried out in various regions of the world, describing the distribution of genotypes in their regions. However, in India, we have studies only on the prevalence of this organism in women of the reproductive age group and in antenatal women [8–11]. The studies on the genotype distribution in India are scarce. The available one dates back to 1976 which in turn is conducted by conventional serotyping methods [12]. Thus, the aim of this study is to find out the genotype distribution of group B *Streptococcus* in various clinical infections and in addition to identify the possible relationships between genotypes, clinical specimen types, and antibiotic resistance profiles.

## 2. Materials and Methods

It was a cross-sectional, descriptive study conducted in the department of microbiology in a tertiary care hospital. The study was approved by the institute ethics committee (IEC/RC/17/29).

### 2.1. Specimen Collection, Processing, and Identification

All consecutive GBS isolates from various clinical specimens during the study period were collected and stocked in Trypticase soy broth at −20°C till further use. The GBS isolates were identified using conventional methods on the basis of colony morphology, Gram staining, catalase, CAMP tests, hippurate hydrolysis, and latex agglutination tests with specific antisera against group B (bioMerieux, France) [13].

### 2.2. Antibiotic Susceptibility Pattern of the Strains Isolated

Antibiotic susceptibility testing was performed by the Kirby–Bauer disk-diffusion method on Mueller–Hinton agar supplemented with 5% sheep blood for the following antimicrobial agents: penicillin (10 U), ampicillin (10 *µ*g), erythromycin (15 *µ*g), clindamycin (2 *µ*g), ofloxacin (5 *µ*g), cefotaxime (30 *µ*g), linezolid (30 *µ*g), and vancomycin (30 *µ*g) (HiMedia Laboratories, Mumbai). The inoculum for susceptibility testing and interpretation was performed as per the Clinical and Laboratory Standards Institute (CLSI) guidelines [14].

### 2.3. PCR for Genotype Identification

All GBS isolates were subjected to multiplex PCR assays targeting nine capsular polysaccharide synthesis (*cps*) genes (Table 1) by using following primers as described by Poyart et al. [15].

The assay consisted of two reaction mixes. The mix (i) has primers for types Ia, Ib, II, IV, and V, and the mix (ii) consists of primers for types III, VI, VII, and VIII. The reagent composition of the mix (i) includes 2.5 *µ*l of extracted bacterial DNA, 1 *µ*l each of forward and reverse primers, and 12.5 *µ*l of master mix preparation. The mix (ii) contained 2.5 *µ*l of extracted bacterial DNA, 1 *µ*l each of forward and reverse primers, 12.5 *µ*l of master mix preparation, and 2 *µ*l of sterile distilled water. The cycling conditions were as follows: 94°C for 3 minutes, followed by 30 cycles of denaturation (94°C, 30 seconds), annealing (58°C, 1 minute), extension (72°C, 1 minute), and final extension (72°C, 5 minutes). PCR products were then electrophoresed on 1.5% agarose gel containing ethidium bromide (GeNei) and visualized by using a gel documentation system (BioRad).

### 2.4. Sequence Analysis

The amplified products were purified and sequenced at Xcelris, Gujarat, India. Nucleotide sequences obtained were edited using Technelysium, DNA sequencing software. The nucleotide sequence was matched with a sequence by the highest maximum identity score in GenBank by performing nucleotide BLAST analysis using the NCBI nucleotide BLAST tool (https://www.ncbi.nlm.nih.gov/BLAST).

### 2.5. Statistical Analysis

All analyses were performed using GraphPad version 3.05. Genotype distribution was described in percentages. Fisher's exact test was used, to find an association between genotypes and samples types as well as between genotypes and antibiotic resistance, with *P* < 0.05 considered to be statistically significant.

## 3. Results

### 3.1. Clinical Details and Bacterial Characteristics

**Figure 1:**
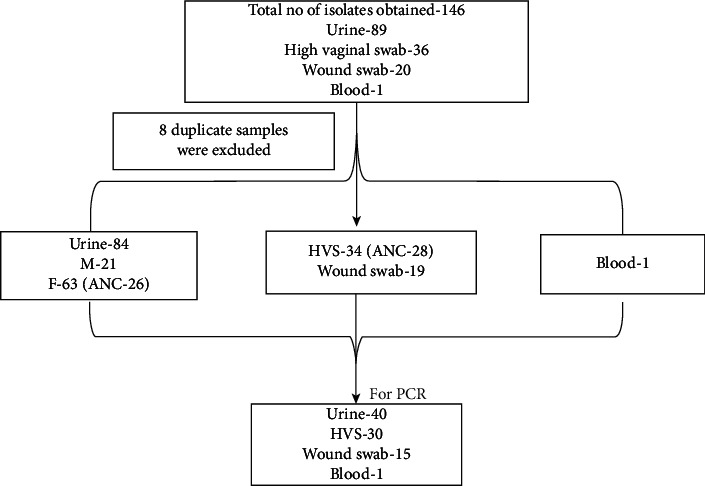
Characteristics of the study population.

A total of 146 GBS isolates were received during the study period (Figure 1). Following the exclusion of 8 duplicate samples, 138 were included in the study from 34 men and 104 women. Of the 138 strains, 84 (60.8%) were isolated from urine, 34 (24.6%) from a high vaginal swab, 19 (13.7%) from skin and soft tissue infections, and 01 (0.7%) from blood samples.

### 3.2. Genotype Distribution

**Figure 2:**
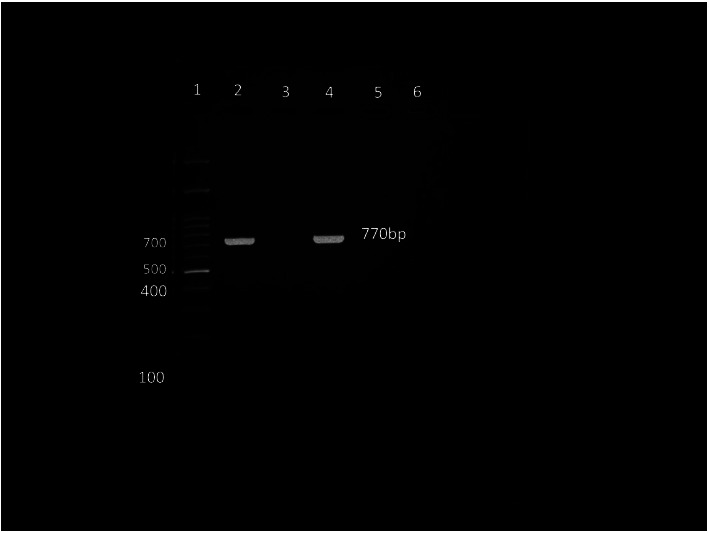
Gel electrophoresis picture of multiplex PCR, showing the positive band for serotype Ib. ^*∗*^Lane 1: ladder 100 bp; lanes 2 and 4: positive samples for serotype Ib; lanes 3, 5, and 6: negative samples.

Among the 138 isolates, 86 were subjected to multiplex PCR for genotype identification. The genotype of the isolates specimen wise is shown in Table 2. A total of five genotypes were identified in this study, the predominant one being genotype Ib (34.9%), followed by III (20.9%), II (16.3%), Ia (12.7%), and V (11.6%) (Figure 2). Three isolates were nontypeable.

### 3.3. Antibiotic Sensitivity Pattern of Isolates

**Figure 3:**
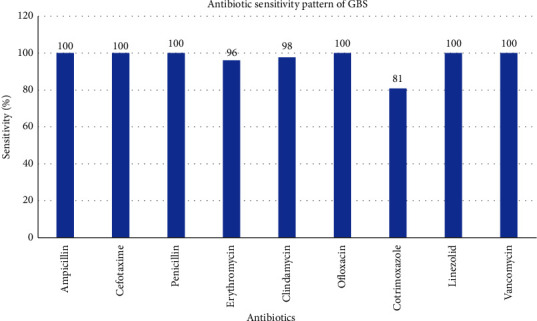
Antibiotic sensitivity pattern of GBS isolates.

All 138 GBS isolates were sensitive to penicillin, cefotaxime, ampicillin, ofloxacin, linezolid, and vancomycin, whereas sensitivity of 91/112 (81) %, 127/132 (96%), and 129/132 (98%) was observed for cotrimoxazole, erythromycin, and clindamycin, respectively (Figure 3). Inducible clindamycin resistance was not observed among these isolates. The serotype distribution and the pattern of antimicrobial resistance among *S.agalactiae* isolates are depicted in Table 3.

### 3.4. Sequencing of the Amplified Products

One strain of genotype III matched the sequence of *Streptococcus agalactiae* capsular locus (cps) operon (serotype III), with accession no LT671986 (Table 4).

## 4. Discussion

*S.agalactiae* has been widely associated with pregnancy-related GBS infections causing serious infections in neonates and was suggested to provide antibiotic prophylaxis based on screening antenatal mothers in the last trimester of pregnancy. However, apart from neonatal infections, GBS has also been reported in multiple invasive infections in adults. Thus, it is believed that the introduction of a vaccine might remain an effective option to reduce GBS infections in the long term. However, due to the existence of multiple serotypes based on capsular antigens, a universally effective vaccine should be introduced, which in turn requires the distribution of common serotypes in different parts of the world. Therefore, with the intention to address the existing knowledge gap on serotype distribution in India, this study was undertaken.

In this study, type Ib was the most common accounting for 34.9% of isolates, followed by types III (20.9%), II (16.3%), Ia (12.7%), and V (11.6%). This finding is similar to that of the Indian study by Prakash et al., depicting serotype Ib as the most predominant type (23.7%), followed by type III (22.7%) [13]. However, serotype distribution widely varies among different countries. Serotype III as the most common has been reported in Chinese infants [16], Japanese neonates [17], Sweden [6], Zimbabwe [18, 19], Europe [20], Hamadan [1],^,^ and Korea [21]. Similarly, serotype V as the predominant type has been reported in Egypt [22], Gambia [23], and central Africa [24]. Studies from Brazil [25] depict Ia as the most common serotype, and serotype II was the most commonly reported in the Thai-Myanmar border of South East Asia [3].

Serotypes VI–IX were not reported in this study. This is similar to most of the studies across the globe, reporting serotypes belonging to I–V, whereas serotypes VI–IX are rarely described. In contrast, serotypes VIII (36%) and VI (25%) were the most common types reported in healthy pregnant women in Japan [26].

While analyzing the correlation between genotypes and clinical specimens, some statistical association was observed between types Ib and III with the vaginal specimen and type II with a urine specimen in the current study. This is in contrast with the other study stating a statistically significant association between type Ia and vaginal colonization and also between type III and urine samples [1]. However, with a smaller number of samples being analyzed in each type, these associations may not be considered clinically significant. Prakash et al. also demonstrated that there was no particular serotype predominant in any of the clinical specimens, except in cases of neonatal septicemia where Ib was the predominant type [12].

Regarding the treatment of group B streptococcal infections, penicillin remains the drug of choice for the majority of cases. Fortunately, penicillin resistance as a problem among GBS has not been reported across the globe or in this study. However, reports of *S. agalactiae* with reduced penicillin susceptibility are being observed in some studies, emphasizing the need for continuous surveillance to guide empirical therapy [27–29]. Alternate drugs like erythromycin and clindamycin come into play whenever an allergy to penicillin arises. The majority of the isolates retain susceptibility to these alternate drugs. However, a few isolates, 4% and 2%, are resistant to erythromycin and clindamycin, respectively (constitutive resistance), in this study. Inducible clindamycin resistance was not observed among these isolates. Also, varying rates of resistance, 3.8%–14.5% and 3%–13% to erythromycin and clindamycin, respectively, are reported by most studies evaluating its susceptibility [4, 5, 20, 24]. Alarming rates varying as high as 92.5% and 87.5% of resistance to erythromycin and clindamycin have also been reported [30]. While there are studies reporting an association between macrolide resistance and serotypes Ib [31], III, and V, no such association was found in this study (Fisher's exact test).

## 5. Conclusion

The current study findings are in line with the reports of most countries worldwide, demonstrating that most of the isolates belong to genotypes I–V. Type Ib was the predominant type reported in this study, followed by types III, II, Ia, and V. The GBS genotype distribution pattern studied adds knowledge to the existing scientific data. However, the results obtained from this study just represent the data from a fraction of the entire population. Therefore, a similar type of study has to be conducted in different regions of the country to know the actual serotype distribution across the country.

## Figures and Tables

**Table 1:** Primer sequences and the gene targets used in the study.

Primer name	Primer sequence (5′ to 3′)	Gene target	Size (bp)	Accession number
Ia-F	GGTCAGACTGGATTAATGGTATGC	*cps1aH*	521 & 1826	LT671983.1
Ia-R	GTAGAAATAGCCTATATACGTTGAATGC	*cps1aH*		
Ib-F	TAAACGAGAATGGAATATCACAAACC	*cps1bJ*	770	LT671984.1
Ib-R	GAATTAACTTCAATCCCTAAACAATATCG	*cps1bK*		
II-F	GCTTCAGTAAGTATTGTAAGACGATAG	*cps2K*	397	LT671985.1
II-R	TTCTCTAGGAAATCAAATAATTCTATAGGG	*cps2K*		
III-F	TCCGTACTACAACAGACTCATCC	*cps1a/2/3I*	1826	LT671986.1
III-R	AGTAACCGTCCATACATTCTATAAGC	*cps1a/2/3J*		
IV-F	GGTGGTAATCCTAAGAGTGAACTGT	*cps4N*	578	LT671987.1
IV-R	CCTCCCCAATTTCGTCCATAATGGT	*cps4N*		
V-F	GAGGCCAATCAGTTGCACGTAA	*cps5O*	701	LT671988.1
V-R	AACCTTCTCCTTCACACTAATCCT	*cps5O*		
VI-F	GGACTTGAGATGGCAGAAGGTGAA	*cps6I*	487	LT671989.1
VI-R	CTGTCGGACTATCCTGATGAATCTC	*cps6I*		
VII-F	CCTGGAGAGAACAATGTCCAGAT	*cps7M*	371	LT671990.1
VII-R	GCTGGTCGTGATTTCTACACA	*cps7M*		
VIII-F	AGGTCAACCACTATATAGCGA	*cps8J*	282	LT671991.1
VIII-R	TCTTCAAATTCCGCTGACTT	*cps8J*		

**Table 2:** Genotype distribution of the *S.agalactiae* strain according to clinical specimens.

Genotype	Urine *n*(%)	High vaginal swab *n*(%)	Wound swab *n* (%)	Blood *n*(%)	Total *n*(%)	*P* value
Ia	8 (20)	—	2 (13.3)	1	11 (12.7%)	0.1033
Ib	10 (25)	16 (53.3)	4 (26.7)	—	30 (34.9%)	**0.0166**
II	11 (28)	2 (6.6)	1 (6.7)	—	14 (16.3%)	**0.0168**
III	3 (7.5)	10 (33.3)	5 (33.3)	—	18 (20.9%)	**0.0041**
V	6 (15)	2 (6.7)	2 (13.3)	—	10 (11.6%)	0.5035
Nontypeable	2 (5)	—	1 (6.7)	—	3 (3.4%)	0.5955
Total	40	30	15	1	86	

Bold values represent the statistically significant association between the genotype and the clinical specimen.

**Table 3:** Serotype distribution and pattern of antimicrobial resistance among S.agalactiae isolates.

Genotype	*N*	Erythromycin *n*(%)	Clindamycin *n*(%)	Cotrimoxazole *n*(%)	*P* value
Ia	11	1 (9.1%)	1 (9.1%)	—	0.4286
Ib	30	—	—	—	—
II	14	1 (7.1%)	—	1 (7.1%)	0.4286
III	18	12 (66.7%)	2 (11.1%)	1 (5.5%)	0.5975
V	10	2 (20%)	—	—	—
Nontypeable	3	—	—	—	—
Total	86	16 (18.6%)	3 (3.5%)	2 (2.3%)	

**Table 4:** Nucleotide sequence of genotype III obtained in the study, showing the NCBI blast results with its identity score and accession number.

Description	Scientific name	Max score	Total score	Query cover (%)	Percentage identity (%)	Acc. len	Accession
*Streptococcus agalactiae* capsular locus (cps) operon (serotype III), strain NCTC11080	*Streptococcus agalactiae*	1615	1615	97	96.73	15760	LT671986.1

## Data Availability

Raw data were generated at the Pondicherry Institute of Medical Sciences, a tertiary care hospital. Derived data supporting the findings of this study are available from the corresponding author on request.
